# microRNA-Based Cancer Diagnosis and Therapy

**DOI:** 10.3390/ijms25010230

**Published:** 2023-12-22

**Authors:** Hexin Chen

**Affiliations:** Department of Biological Sciences, University of South Carolina, Columbia, SC 29208, USA; hchen@biol.sc.edu; Tel.: +1-803-777-2928

MicroRNAs (miRNAs) are small non-coding RNA molecules that regulate gene expression post-transcriptionally by impeding mRNA translation or stability. Dysregulated miRNA expression has been implicated across tumor onset, growth, recurrence, and therapeutic resistance [1]. Their capacity for simultaneously modulating diverse cellular pathways has elicited interest in miRNAs as novel diagnostic markers and treatment modalities for cancer. However, this multi-targeting potential represents both therapeutic opportunity and possible risks. miRNA-directed inhibition of myriad targets could overcome intra-tumor heterogeneity and diverse drug resistance mechanisms, while coordinated suppression of tumor cell expansion alongside microenvironmental signaling manipulation demonstrates their multifaceted regulatory roles. Conversely, promiscuous gene suppression may inadvertently trigger off-target effects. Therefore, ongoing research aims to capitalize on the far-reaching regulatory functions of miRNAs for selective and synergistic anticancer activity while avoiding toxicity. This Special Issue attempts to collect the most recent progress made by multidisciplinary researchers in tackling the key challenges in microRNA-based cancer diagnosis and treatment.

Dysregulated miRNAs in cancers can serve as diagnostic and prognostic biomarkers [2,3]. Extensive profiling of microRNA (miRNA) expression patterns in tumorous and adjacent non-tumor tissues has yielded abundant miRNA signatures with potential prognostic and therapeutic implications [4]. Beyond evaluating tumor-derived miRNAs, those circulating freely or inside extracellular vesicles in liquid biopsies could serve as minimally invasive biomarkers for early detection or treatment monitoring [5,6,7,8]. Moreover, rapidly advancing bioinformatics facilitates elucidation of the intricate connections between miRNA profiles and downstream oncogenic targets, activated signaling pathways, patient survival, and drug response dynamics. Consolidation of expansive miRNA datasets with artificial intelligence and machine learning could eventually transform these patterns into comprehensive precision medicine tools complementing clinic-based decisions [9,10]. Overall, multi-level miRNA analyses—spanning identification in cells and circulation to association mapping via algorithmic learning—show promise for improving cancer management and care through enhanced risk stratification, therapeutic matching, and non-invasive disease tracking.

Dysregulated microRNAs in cancer also serve critical functions in governing tumorigenesis and therapeutic sensitivity [11]. Depending on the tissue context, individual miRNAs exhibit tumor-suppressive or oncogenic activities. Downregulation of tumor suppressor miRNAs enables overexpression of proto-oncogenes, while upregulation of onco-miRs suppresses tumor suppressor genes [11]. Furthermore, dysregulated miRNAs influence drug resistance pathways including efflux pumps and signaling cascades regulating the cell cycle, proliferation, and apoptosis like JAK/STAT, MAPK, PI3K/AKT, WNT, and hedgehog [12]. Elucidating specific miRNAs that promote immune evasion, chemotherapy resistance, or anti-tumor immunity could inform improved outcomes [13]. miRNA-based therapeutics have emerged owing to favorable pharmacokinetic properties and enhanced efficacy/safety over traditional regimens. Earlier therapeutic approaches utilize anti-miRs, miRNA mimics, or miRNA-encoding vectors to directly modify miRNA expression. With a large amount of supporting data generated from in vitro, in vivo, and pre-clinical studies during the past two decades or so, several miRNA therapies have recently entered clinical testing. Phase I trials of miRNA-34 [14], miRNA-155 [15], and miRNA-16 [16] mimics demonstrated therapeutic benefit against liver, lymphoma, melanoma, and/or lung cancers. Overall, directly targeting dysregulated oncogenic or tumor-suppressive miRNAs represents a clinically viable approach to improve cancer prognosis and treatment response through multiple underlying mechanisms.

To further advance miRNA-directed therapies, alternative treatment approaches have emerged through preclinical development and evaluation. One strategy utilizes bioinformatically designed, pharmacologically active small molecules that permeate cells and bind to specific miRNA structural motifs or biogenesis machinery components [17]. Another approach deploys miRNA “sponges”—RNA constructs harboring multiple binding sites to sequester and suppress select miRNAs [18]. For example, circular RNA sponges represent an emerging technology leveraged for breast cancer inhibition through miR-550a binding and functional blockade [19]. Overall, while direct manipulation of dysregulated miRNAs shows early clinical promise, complementary targeting of regulatory processes governing their biogenesis and activity could provide additional options to fine-tune expression. By interceding at multiple points along the miRNA regulatory cascade, these innovative tools aim to potentiate and diversify the therapeutic potential of oncogenic or tumor suppressor miRNA networks for improved cancer prognosis and survival.

In addition to monotherapy applications, miRNA-based treatments can synergistically augment conventional chemotherapy and immunotherapy responses [20]. Co-administration of miRNAs alongside anticancer agents cooperatively disables key oncogenic pathways to amplify anti-tumor effects, as evidenced by enhanced triple-negative breast cancer suppression without added toxicity via coordinated TCF-7 gene silencing and doxorubicin delivery [21]. Immunotherapy combinations also demonstrate vast potential to expand the clinical benefit of immune checkpoint inhibitors. Though efficacious for some patients, checkpoint blockade monotherapies risk potentially fatal immune-related adverse events and intrinsically subvert only a subset of tumor evasion mechanisms [22]. miRNAs could potentiate checkpoint inhibition while attenuating hyperimmune reactions owing to their multifaceted targeting capabilities. For example, miR143 can dually inhibit immune checkpoint genes B7-H3/B7-H4 and angiogenesis pathways [23,24]. Ongoing investigations thus seek to identify optimal miRNA partners for checkpoint inhibitors that elicit cooperative anti-tumor immune responses while preventing toxicity. In summary, miRNAs represent versatile adjuncts to complement conventional cytotoxic and immunotherapeutic modalities through synergistic, broad-based molecular targeting.

The emerging role of microRNAs (miRNAs) in cancer drug resistance provides valuable insights into overcoming treatment obstacles and improving outcomes [25]. However, translating these small non-coding RNA molecules into clinical practice still faces significant hurdles. Their dysregulation plays a part in chemoresistance across cancer types, and targeting miRNAs represents a promising therapeutic approach to disable molecular pathways underlying progression and resistance. Specific applications include utilizing miRNA mimics or inhibitors to regulate gene expression and overcome chemoresistance, developing miRNA biomarkers to predict drug response, and employing miRNA profiles for early diagnostic detection of resistant tumors. Still, efficient targeted delivery poses a key challenge due to rapid bloodstream degradation and poor penetration into cells. The capability of miRNAs to concurrently suppress multiple genes also leads to possible adverse events. Advancing delivery methods through viral vectors, nanoparticles, or exosomes could enable precision miRNA augmentation of chemotherapy, radiation, and immunotherapy. However, expanded understanding of selective miRNA–gene interactions for particular cancer types would allow optimization of structures and doses to increase efficiency while avoiding side effects. Overall, miRNAs show potential for overcoming the pressing problem of cancer drug resistance, but clinical translation awaits solutions for safe and specific cellular delivery along with elucidation of miRNA pathway dynamics.

## References

[1] Rupaimoole R., Slack F.J. (2017). MicroRNA therapeutics: Towards a new era for the management of cancer and other diseases. Nat. Reviews. Drug Discov..

[2] Shademan B., Karamad V., Nourazarian A., Masjedi S., Isazadeh A., Sogutlu F., Avcı C.B. (2023). MicroRNAs as Targets for Cancer Diagnosis: Interests and Limitations. Adv. Pharm. Bull..

[3] Wang J., Chen J., Sen S. (2016). MicroRNA as Biomarkers and Diagnostics. J. Cell. Physiol..

[4] Hu Y., Dingerdissen H., Gupta S., Kahsay R., Shanker V., Wan Q., Yan C., Mazumder R. (2018). Identification of key differentially expressed MicroRNAs in cancer patients through pan-cancer analysis. Comput. Biol. Med..

[5] He Y., Deng F., Yang S., Wang D., Chen X., Zhong S., Zhao J., Tang J. (2017). Exosomal microRNA: A novel biomarker for breast cancer. Biomark Med..

[6] Hamam R., Hamam D., Alsaleh K.A., Kassem M., Zaher W., Alfayez M., Aldahmash A., Alajez N.M. (2017). Circulating microRNAs in breast cancer: Novel diagnostic and prognostic biomarkers. Cell Death Dis..

[7] Shin V.Y., Siu J.M., Cheuk I., Ng E.K., Kwong A. (2015). Circulating cell-free miRNAs as biomarker for triple-negative breast cancer. Br. J. Cancer.

[8] Chen M., Calin G.A., Meng Q.H. (2014). Circulating microRNAs as Promising Tumor Biomarkers. Adv. Clin. Chem..

[9] Azari H., Nazari E., Mohit R., Asadnia A., Maftooh M., Nassiri M., Hassanian S.M., Ghayour-Mobarhan M., Shahidsales S., Khazaei M. (2023). Machine learning algorithms reveal potential miRNAs biomarkers in gastric cancer. Sci. Rep..

[10] Lopez-Rincon A., Mendoza-Maldonado L., Martinez-Archundia M., Schönhuth A., Kraneveld A.D., Garssen J., Tonda A. (2020). Machine Learning-Based Ensemble Recursive Feature Selection of Circulating miRNAs for Cancer Tumor Classification. Cancers.

[11] Esquela-Kerscher A., Slack F.J. (2006). Oncomirs—MicroRNAs with a role in cancer. Nat. Rev. Cancer.

[12] Alsaab H.O., Abdullaev B., Alkhafaji A.T., Alawadi A.H., Jahlan I., Bahir H., Bisht Y.S., Alsaalamy A., Jabbar A.M., Mustafa Y.F. (2023). A comprehension of signaling pathways and drug resistance; an insight into the correlation between microRNAs and cancer. Pathol. Res. Pract..

[13] Nail H.M., Chiu C.C., Leung C.H., Ahmed M.M.M., Wang H.D. (2023). Exosomal miRNA-mediated intercellular communications and immunomodulatory effects in tumor microenvironments. J. Biomed. Sci..

[14] Beg M.S., Brenner A.J., Sachdev J., Borad M., Kang Y.K., Stoudemire J., Smith S., Bader A.G., Kim S., Hong D.S. (2017). Phase I study of MRX34, a liposomal miR-34a mimic, administered twice weekly in patients with advanced solid tumors. Investig. New Drugs.

[15] Witten L., Slack F.J. (2020). miR-155 as a novel clinical target for hematological malignancies. Carcinogenesis.

[16] van Zandwijk N., Pavlakis N., Kao S.C., Linton A., Boyer M.J., Clarke S., Huynh Y., Chrzanowska A., Fulham M.J., Bailey D.L. (2017). Safety and activity of microRNA-loaded minicells in patients with recurrent malignant pleural mesothelioma: A first-in-man, phase 1, open-label, dose-escalation study. Lancet. Oncol..

[17] Zhao R., Fu J., Zhu L., Chen Y., Liu B. (2022). Designing strategies of small-molecule compounds for modulating non-coding RNAs in cancer therapy. J. Hematol. Oncol..

[18] Peña-Paladines J.J., Wong C.H., Chen Y. (2023). Circularized RNA as novel therapeutics in cancer. Int. J. Biochem. Cell Biol..

[19] Meng L., Chang S., Sang Y., Ding P., Wang L., Nan X., Xu R., Liu F., Gu L., Zheng Y. (2022). Circular RNA circCCDC85A inhibits breast cancer progression via acting as a miR-550a-5p sponge to enhance MOB1A expression. Breast Cancer Res. BCR.

[20] Chitkara D., Singh S., Mittal A. (2016). Nanocarrier-based co-delivery of small molecules and siRNA/miRNA for treatment of cancer. Ther. Deliv..

[21] Gong C., Tian J., Wang Z., Gao Y., Wu X., Ding X., Qiang L., Li G., Han Z., Yuan Y. (2019). Functional exosome-mediated co-delivery of doxorubicin and hydrophobically modified microRNA 159 for triple-negative breast cancer therapy. J. Nanobiotechnology.

[22] Ribas A., Wolchok J.D. (2018). Cancer immunotherapy using checkpoint blockade. Science.

[23] Qian X., Yu J., Yin Y., He J., Wang L., Li Q., Zhang L.-Q., Li C.-Y., Shi Z.-M., Xu Q. (2013). MicroRNA-143 inhibits tumor growth and angiogenesis and sensitizes chemosensitivity to oxaliplatin in colorectal cancers. Cell Cycle.

[24] Zhou X., Mao Y., Zhu J., Meng F., Chen Q., Tao L., Li R., Fu F., Liu C., Hu Y. (2016). TGF-β1 promotes colorectal cancer immune escape by elevating B7-H3 and B7-H4 via the miR-155/miR-143 axis. Oncotarget.

[25] Chen B., Dragomir M.P., Yang C., Li Q., Horst D., Calin G.A. (2022). Targeting non-coding RNAs to overcome cancer therapy resistance. Signal Transduct. Target. Ther..

